# Case Report: A case of disseminated cutaneous listeriosis following appendicitis from Lao PDR

**DOI:** 10.12688/wellcomeopenres.20210.2

**Published:** 2024-05-15

**Authors:** Terry John Evans, Vannavong Siratana, Timothy Venkatesan, Viengmon Davong, Khamphong Thanadabouth, Elizabeth A. Ashley

**Affiliations:** 1 Lao-Oxford-Mahosot Hospital-Wellcome Trust Research Unit, Mahosot Hospital, Vientiane, Lao People's Democratic Republic; 2 London School of Hygiene and Tropical Medicine, London, UK; 3 Microbiology Laboratory, Mahosot Hospital, Vientiane, Lao People's Democratic Republic; 4 Intensive Care Unit, Mahosot Hospital, Vientiane, Lao People's Democratic Republic; 5 Centre for Tropical Medicine and Global Health, Nuffield Department of Medicine, University of Oxford, Oxford, England, UK

**Keywords:** Listeria, food-borne, bacteraemia, meningitis, pustular rash, sarcoidosis, southeast Asia, Laos

## Abstract

**Background:**

*Listeria monocytogenes* is a food-borne pathogen that is a rare cause of bacteraemia and meningitis in immunosuppressed patients, and carries a high mortality rate. Cutaneous manifestations of listeriosis are rare, and are usually associated with direct inoculation of the skin.

**Case:**

A 41-year-old woman who initially presented to a hospital in Laos with appendicitis was diagnosed with disseminated listeriosis with cutaneous involvement. Intra-abdominal pathology probably contributed to bacterial bloodstream invasion. Initial treatment with meropenem was switched to ampicillin based on best practice, however our patient died 5 days after diagnosis.

**Conclusions:**

This case highlights listeriosis as an important cause of mortality in low- and middle-income countries, exacerbated by poor availability of laboratory diagnostics and ineffective empiric antibiotic regimens. Improvements in food hygiene, surveillance, and increased laboratory capacity are important strategies to reduce rates of infection and clinical outcomes.

## Introduction

Listeriosis is a food-borne infection caused by the bacterium *Listeria monocytogenes*, manifesting as a wide spectrum of clinical disease, typically ranging from mild gastrointestinal upset to bacteraemia and meningo-encephalitis in susceptible hosts ^1^. The global burden of listeriosis is insufficiently described, particularly in low- and middle-income countries (LMICs) ^2^. Early recognition of *L. monocytogenes* infection is important as it is not optimally treated by most empirical antibiotic regimens for bacterial sepsis, and invasive infections have a high mortality rate of 20-40% ^3– 5^. Increasing clinicians’ knowledge of the patterns of disease caused by *L. monocytogenes* is therefore critical.

Although recognised in approximately 10% of invasive neonatal infections ^6^, cutaneous manifestations of listeriosis in adults are rare. A review of prior case reports found most adult cases were caused by direct inoculation of the skin, either in farmers or veterinarians who had exposure to animal products of conception. Accordingly, skin lesions were localised to the site of inoculation. Cutaneous disease from haematogenous spread was infrequent and associated with severe immunocompromise, including haematological malignancy and HIV ^7^.

We report a case of disseminated cutaneous listeriosis following appendicitis in a female patient diagnosed with sarcoidosis and taking prednisolone. This case reinforces the potential for haematogenous spread as a cause of disseminated cutaneous listeriosis.

## Case report

We report the case of a 41-year-old Lao woman who lived in Vientiane Capital, Laos. She was diagnosed with sarcoidosis two years prior to presentation, although details of the diagnostic work-up were unavailable. She had no other notable past medical history apart from early menopause aged 39 years old.

One month before her admission with appendicitis, she was admitted to our hospital with cough and dyspnoea. A thoracic computerised tomography (CT) scan demonstrated a mild-to-moderate left-sided pleural effusion alongside widespread peribronchovascular honeycombing and hilar-mediastinal lymphadenopathy; this was diagnosed as stage IV sarcoidosis. Investigations for tuberculosis were not performed. She remained in hospital for 2 weeks, and on discharge was prescribed prednisolone 40 mg daily to control a sarcoid flare.

She re-presented 2 weeks later with a five-day history of worsening abdominal pain in the right upper and lower quadrants, and diarrhoea. On admission she was afebrile, with unremarkable vital signs (blood pressure 110/80 mmHg, heart rate 74 bpm, peripheral oxygen saturations 98% on room air, and respiratory rate 20/min). An abdominal ultrasound scan demonstrated hepatomegaly; the appendix was not described. She was diagnosed clinically with appendicitis and taken for surgery the same day. Intra-operative findings included peritonitis, copious amounts of pus and a caecal mass (10 x 10 cm). She underwent a hemicolectomy with primary ileo-colic anastomosis. The caecal mass was excised and sent for histopathological examination, and showed suppurative appendicitis.

**Figure 1.**
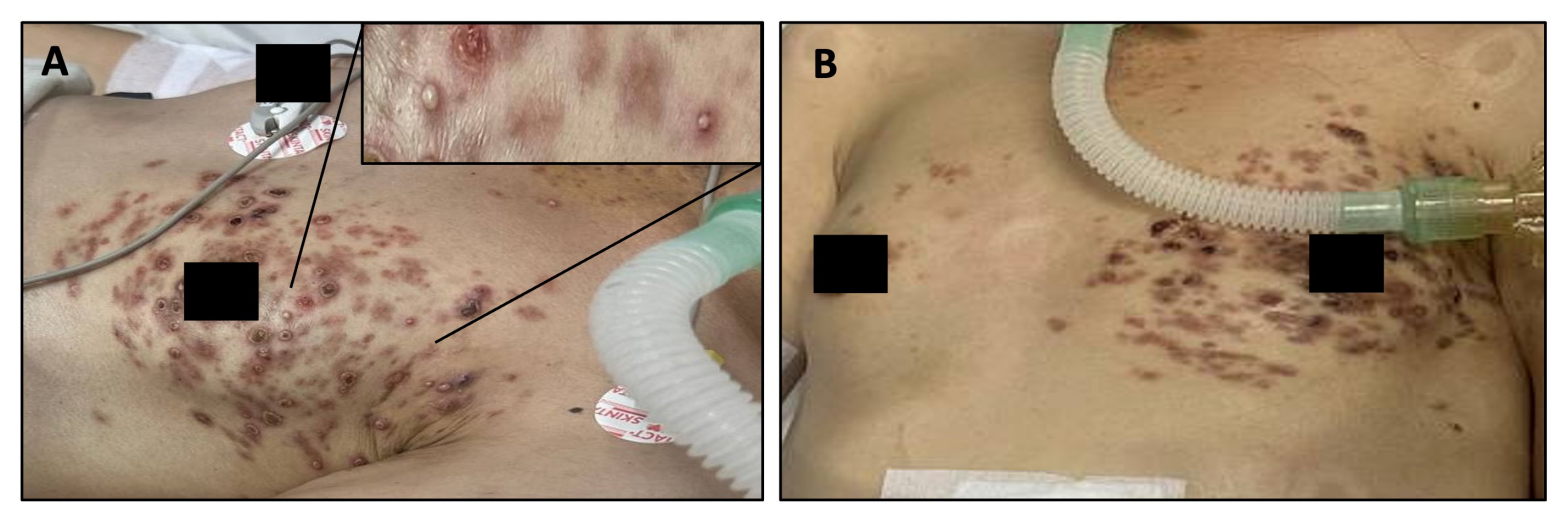
**A**: Disseminated papulo-pustular rash concentrated on the left chest. Inset: A cluster of lesions with pustules at a variety of stages. **B**: Initial resolution of the rash, with improvement in the pustules.

Post-operatively, she was treated with ceftriaxone (2 g intravenously, every 24 hours), gentamicin (5 mg/kg intravenously, once daily) and metronidazole (500 mg intravenously, every 8 hours), and she initially improved. However, a florid, disseminated pustular rash developed the following day, initially across her left chest, before spreading to her upper thighs and lower abdomen ( Figure 1A). The rash consisted of numerous small, well-demarcated erythematous lesions of varying sizes, many with pustules ( Figure 1A, inset). On direct questioning, the patient’s family revealed, surprisingly, that a milder version of this rash had first appeared on the patient’s arms and legs two months earlier, although it is unclear if it truly had the same appearance and aetiology. Of note, a biopsy of one of these lesions was taken prior to this admission, in connection with her admission for presumed sarcoidosis. The skin biopsy demonstrated a nodular infiltrate in the dermis, composed of a histiocytic aggregation and neutrophilic micro-abscesses admixed with plasma cells, and multinucleated giant cells. No sarcoid granulomas or bacteria were seen. The histopathology report commented that the observed mixed cell granulomas can be associated with chronic granulomatous infections; notably, these findings are consistent with listeriosis.

On the fourth post-operative day, the patient deteriorated with septic shock and respiratory failure, with a blood pressure of 80/50 mmHg, respiratory rate of 38/minute and peripheral oxygen saturations of 85% on room air. She was transferred to the Intensive Care Unit for inotropic and vasopressor support with noradrenaline, and was intubated 3 days later due to further respiratory deterioration. Of note, prior to intubation, the patient did not exhibit any signs of meningitis. The antibiotics were escalated empirically to meropenem (1 g intravenously, every 8 hours) and a blood culture was sent, along with a bacterial swab of the pustular rash.

The blood culture flagged positive after 18 hours, with Gram-positive rods seen on Gram stain, subsequently identified as *Listeria monocytogenes* by MALDI-ToF. At the same time, *L. monocytogenes* also grew from the pus swab, confirming a diagnosis of disseminated listeriosis. Initially, meropenem was continued to provide ongoing broad-spectrum coverage given the recent intra-abdominal pathology. Two days after *L. monocytogenes* was isolated, the antibiotic was switched to high-dose intravenous ampicillin (2 g, 6 times daily), which was continued for the remainder of the admission. The isolates from both blood and pus were sensitive to penicillin, meropenem, co-trimoxazole and erythromycin, as determined by disk diffusion testing ( https://www.eucast.org).

The appearance of the rash improved, with some resolution of the pustules, but unfortunately, the patient’s clinical condition was unchanged, with on-going cardiorespiratory failure. Given the lack of clinical improvement, the severity of the infection, and the poor prognosis of invasive listeriosis, after one week of intensive care treatment, the decision was taken by the patient’s family to stop active treatment. It was arranged for her to be taken home for end-of-life care.

## Discussion

This patient was diagnosed with listeriosis with dissemination to the skin, and this was associated with appendicitis. We could find only one other case where listeriosis was associated with appendicitis ^8^ although cases complicating a range of other intra-abdominal pathologies have been reported ^9^. Appendicitis in this case was likely caused by *Listeria* infection, which to our knowledge has not been described before.

No microbiological samples were sent on admission or intra-operatively, and therefore it is not known when our patient developed invasive disease. Neither is it possible to say with certainty when the patient ingested *Listeria* due to the incubation period that can exceed 30 days ^5^. Our patient was diagnosed with listeriosis on day 5 of her admission, and although hospital-acquired outbreaks are well documented, we have not noted any other cases in our hospital.

Although the vesiculo-pustular rash was disseminated, it was concentrated in several discrete dermatomes, such as the left T4 dermatome as shown in Figure 1. This initially raised the possibility of super-infected shingles. However, lesions were bilateral, which was more clearly appreciated on the thighs and lower abdomen. Viral swabs of the vesicles were not taken, however cultures confirmed *Listeria* and retrospective testing demonstrated the patient was not viraemic with varicella-zoster virus at the time the positive blood culture was taken, making shingles unlikely.

A lumbar puncture was not performed during admission and so central nervous system infection by *L. monocytogenes* was not excluded, although the patient never displayed symptoms suggestive of meningitis. However, the dose of ampicillin selected was adequate to cover the possibility of meningo-encephalitis ( *i.e.*, 2 g intravenously, every 4 hours).

Finally, regarding the clinical presentation, it is important to consider the possibility that the bilateral pneumonia, pleural effusion and lymphadenopathy described above that were attributed to a sarcoid flare may in fact have been due to progression of chronic listeriosis.

Several previously identified risk factors for severe disseminated disease were present in this case. First, the integrity of the patient’s colonic mucosa was impaired due to appendicitis. Second, treatment with broad-spectrum antibiotics removed the protective effects of the normal gut microbiota ^10^. Third, the patient had been receiving high-dose oral corticosteroids, and although these were stopped prior to admission, glucocorticoids are a risk factor for invasive listeriosis and strongly associated with fatal outcomes ^11^; they likely accelerated the fulminant course of this infection. Finally, the patient may have been immunosuppressed to some extent by her sarcoidosis. Sarcoidosis is associated with peripheral lymphopenia, including depressed CD4 counts, although not to the extent seen in advanced HIV infection ^12^. This may affect T-cell-mediated immunity, which is important in the response to *Listeria* infection. Together, these factors may have resulted in a large and overwhelming bacterial burden.

Ampicillin or amoxicillin (2 g intravenously, every 4–6 hours, usually for at least 2 weeks for bloodstream infection, and at least 3 weeks for meningitis) is the first-choice antibiotic for listeriosis, usually in combination with gentamicin due to synergistic action observed *in vitro*. In this case, the preceding intra-abdominal pathology necessitated broad-spectrum antibiotic therapy, and precluded an immediate antibiotic switch from meropenem to ampicillin, which occurred 48 hours after *Listeria* was first isolated. Meropenem has *in vitro* activity against *Listeria*, and features on some treatment guidelines at high dose (2 g intravenously, every 8 hours; John Hopkins ABX guide). There is no data from controlled trials comparing meropenem to ampicillin. However, a Danish retrospective observational study showed that treatment with meropenem was associated with a higher 30-day mortality rate (25% compared to 11% for those treated with penicillins; odds ratio 0.4) – but the impact of confounding factors in this retrospective review cannot be accounted for ^13^. While there are indeed case reports of treatment failure with meropenem ^14^, there are also reports of patient recovery only after a switch to meropenem ^15^. The consensus remains that listeriosis is most securely treated with ampicillin or amoxicillin. Dual therapy with amoxicillin and broad-spectrum agent may a role – either for listeriosis alone, or to simultaneously treat listeriosis and other infections ^16^.

The risk of listeriosis in low- and middle-income countries may be increased due to less access to reliable refrigeration and microbiological food safety monitoring. A recent systematic review of *Listeria* contamination in food, environmental and animal sources in southeast Asia found that up to 43% of food samples in Malaysia, 15% in Thailand, and 16% in Indonesia were affected, with an overall prevalence in southeast Asia of 16% ^17^. A global review of listeriosis concluded that urgent efforts were needed to fill the knowledge gaps concerning the burden of listeriosis in low- and middle-income countries ^2^. No incidence data for the southeast Asia region was found at all. To our knowledge, there are no studies of the prevalence of *Listeria* contamination of food or the environment, or of clinical listeriosis in Laos. However, in our laboratory alone, we have isolated *L. monocytogenes* from seven patients since 2017: in addition to the patient reported here, two patients had *L. monocytogenes* isolated from both blood cultures and CSF, and four had *L. monocytogenes* isolated from blood cultures only. The patients’ ages ranged from 0 to 90 years old – pointing to a diverse case mix in our setting.

## Conclusions

To conclude, listeriosis is likely an under-recognised condition in LMICs, and the commonly used cephalosporin-based empiric antibiotic regimens are not active against it. This underscores the ongoing need to build microbiology diagnostic capacity. Dissemination with cutaneous presentations may follow apparently subtle immunosuppression, and may be associated with significant intra-abdominal pathology.

## Public and patient involvement

There was no formal patient or public involvement in the design or conduct of this work.

## Consent

Written informed consent for publication of their clinical details and clinical images was obtained from the relative of the patient.

## Data Availability

All data underlying the results are available as part of the article and no additional source data are required.
