# Supplementary material for: Case Report: A case of disseminated cutaneous listeriosis following appendicitis from Lao PDR
Source: Wellcome Open Res. 2024 May 15;8:504. Originally published 2023 Nov 7. [Version 2] doi: 10.12688/wellcomeopenres.20210.2 (PMC10905163; doi:10.12688/wellcomeopenres.20210.2)
Supplement: Peer review history

# Reviewer report: Reviewer response for version 2

**DOI:** 10.21956/wellcomeopenres.24013.r83372

**Published:** 2024-05-23

**Authors:** Bruno Hassunuma Carneiro

**Affiliations:** 1 Federal University of Paraná, Curitiba, Brazil

No further suggestions to make.

Are enough details provided of any physical examination and diagnostic tests, treatment given and outcomes?

Yes

Is the case presented with sufficient detail to be useful for other practitioners?

Yes

Is sufficient discussion included of the importance of the findings and their relevance to future understanding of disease processes, diagnosis or treatment?

Partly

Is the background of the case’s history and progression described in sufficient detail?

Partly

Reviewer Expertise:

Internal Medicine and Infectious Diseases

I confirm that I have read this submission and believe that I have an appropriate level of expertise to confirm that it is of an acceptable scientific standard.

# Reviewer report: Reviewer response for version 1

**DOI:** 10.21956/wellcomeopenres.22379.r74093

**Published:** 2024-04-29

**Authors:** Bruno Hassunuma Carneiro

**Affiliations:** 1 Federal University of Paraná, Curitiba, Brazil

The article is very well-written as it illustrates a severe case of invasive listeriosis presenting with cutaneous manifestations after hematogenous spread in the context of a complicated intra-abdominal infection.

 I would not say the patient’s background had no recognized risk factors for invasive listeriosis. In fact, she had received high-dose glucocorticoids for recently diagnosed sarcoidosis. In a large, nationwide prospective study (MONALISA Study), glucocorticoid use was not only a risk factor for invasive listeriosis but also strongly associated with mortality in patients with bacteremia and neurolisteriosis ^1^.

 The case presentation includes details of the investigation, showcases the cutaneous lesions mimicking Herpes Zoster and the presence of *Listeria monocytogenes* recovered from blood and pus cultures. It is important to note that, from a pharmacological standpoint, noradrenaline has some inotropic activity, as cited by the authors; however, its main use in septic shock is as a vasopressor ^2^.

 The authors provide the rationale for the treatment and the employed diagnostic methods very clearly. The patient was treated with broad-spectrum antibiotic regimens to cover intra-abdominal bacteria. As mentioned, gentamicin has historically been used as an adjuvant to treat invasive listeriosis, usually in combination with an aminopenicillin, and meropenem has also been reported to be an effective strategy in some cases. In this particular scenario, to maintain broad-spectrum activity against intra-abdominal pathogens and at the same time include a first-line choice for *Listeria monocytogenes*, the combination regimen of meropenem plus ampicillin could be considered, as this regimen has been previously used for complicated listeriosis. Although the experience with this combination is limited to case reports and has had mixed results, it is worth mentioning ^3^.

 The authors precisely conclude that listeriosis is probably underrecognized and that most broad-spectrum antibiotic regimens that include cephalosporins are not active against *Listeria monocytogenes, *a deadly combination of factors that add up to the complexity of the management of intra-abdominal infections and immunocompromised patients.

Are enough details provided of any physical examination and diagnostic tests, treatment given and outcomes?

Yes

Is the case presented with sufficient detail to be useful for other practitioners?

Yes

Is sufficient discussion included of the importance of the findings and their relevance to future understanding of disease processes, diagnosis or treatment?

Partly

Is the background of the case’s history and progression described in sufficient detail?

Partly

Reviewer Expertise:

Internal Medicine and Infectious Diseases

I confirm that I have read this submission and believe that I have an appropriate level of expertise to confirm that it is of an acceptable scientific standard, however I have significant reservations, as outlined above.

## References

[1] : Clinical features and prognostic factors of listeriosis: the MONALISA national prospective cohort study. *Lancet Infect Dis* .2017;17(5) : 10.1016/S1473-3099(16)30521-7 510-519 10.1016/S1473-3099(16)30521-7 28139432

[2] : Surviving sepsis campaign: international guidelines for management of sepsis and septic shock 2021. *Intensive Care Med* .2021;47(11) : 10.1007/s00134-021-06506-y 1181-1247 10.1007/s00134-021-06506-y 34599691 PMC8486643

[3] : Brain abscesses caused by Listeria monocytogenes in a patient with myasthenia gravis - Case report and systematic review. *Infez Med* .2023;31(4) : 10.53854/liim-3104-16 570-574 10.53854/liim-3104-16 38075415 PMC10705864

# Reviewer report: Reviewer response for version 1

**DOI:** 10.21956/wellcomeopenres.22379.r71633

**Published:** 2024-03-04

**Authors:** Gauri Godbole

**Affiliations:** 1 United Kingdom Health Security Agency, Gastrointestinal Pathogens and Food Safety (One Health) Division, London, UK

This a very nicely written case report showcasing the various complications of listeriosis in an immunosuppressed adult.

 Case report:

 The case is chronologically well presented, with adequate detail, some additional parameters such as white cell count, CRP during her first admission (paragraph 2) should be described, did she have fever? (not mentioned, worth mentioning if she didn't have fever) was she treated with antibiotics for the chest infection during that admission? which ones?

 Regarding the second admission, was the antibiotic susceptibility of the *Listeria monocytogenes *checked (as there is some drug resistance in LMIC) and was synergistic gentamicin given? If it wasn't, is there a reason for omission?

 Discussion:

 It is well presented, T cell dysfunction associated with increased risk of listeriosis is described. The outcome studies for meropenem are appropriately referenced. 

 Conclusion:

 After reading this report I have concluded that this patient had chronic listeriosis which clinically manifested 2 months prior to admission as a skin rash (the later biopsy confirmed classical features seen in Listeriosis such as micro abscesses and mixed cell granulomas not typical of sarcoid. Were any bacteria seen on histology? The bilateral pneumonia, lymphadenopathy and pleural effusions are all consistent but a rare presentation of disseminated listeriosis which was progressing as was untreated. Hey, were mistaken for a sarcoid flare and t's important to draw that out in the discussion.

 The patient was further immunosuppressed with prednisolone which will have rapidly accelerated the fulminant course and dissemination. Involvement of various parts of intestinal tract, the appendix and caecum and peritoneum are not a surprising, cutaneous manifestations are a feature of widespread dissemination and are not due to appendicitis, all compounded by absence of appropriate antibiotics.

 The title of the case report should be changed to disseminated chronic listeriosis with widespread cutaneous manifestations in an immunocompromised adult from Lao PDR

 Overall comments:

 This is an important case report raise awareness of this pathogen in LMIC. The authors highlight lack of laboratory facilities which are extremely important for diagnosis and prevention of poor outcomes. It highlights 2 rare presentations of Listeria in immunocompromised adults- skin manifestations without occupational inoculation and chronic course of illness with widespread pulmonary and gastrointestinal involvement including appendicitis and peritonitis.

Are enough details provided of any physical examination and diagnostic tests, treatment given and outcomes?

Partly

Is the case presented with sufficient detail to be useful for other practitioners?

Yes

Is sufficient discussion included of the importance of the findings and their relevance to future understanding of disease processes, diagnosis or treatment?

Yes

Is the background of the case’s history and progression described in sufficient detail?

Partly

Reviewer Expertise:

Gastrointestinal bacteria, One health AMR and parasitology

I confirm that I have read this submission and believe that I have an appropriate level of expertise to confirm that it is of an acceptable scientific standard, however I have significant reservations, as outlined above.

# Reviewer report: Reviewer response for version 1

**DOI:** 10.21956/wellcomeopenres.22379.r74088

**Published:** 2024-02-20

**Authors:** Ioana Diana Olaru

**Affiliations:** 1 Department of Clinical Research, University of Münster, Münster, Germany

The authors report on a case of disseminated listeriosis in an immunosuppressed patient. The manuscript is well written and relevant for clinical practice. The report is particularly important for LMIC where bacterial identification can be challenging. I have only a few minor comments

 1. Do you have any laboratory results for haematology and chemistry 

 2. Can you comment whether anyone else from the household/ contacts may have suffered from a mild form of infection

Are enough details provided of any physical examination and diagnostic tests, treatment given and outcomes?

Yes

Is the case presented with sufficient detail to be useful for other practitioners?

Yes

Is sufficient discussion included of the importance of the findings and their relevance to future understanding of disease processes, diagnosis or treatment?

Yes

Is the background of the case’s history and progression described in sufficient detail?

Yes

Reviewer Expertise:

Infectious diseases & microbiology, antimicrobial resistance, diagnostics in LMIC

I confirm that I have read this submission and believe that I have an appropriate level of expertise to confirm that it is of an acceptable scientific standard.

# Response

**Published:** 2024-02-29

**Authors:** Terry Evans

**Affiliations:** Nuffield Department of Medicine, University of Oxford, Oxford, England, UK

The authors appreciate the reviewer's careful and critical reading of our case. In this case, there was no history of unwell contacts. Patients' medical records are not computerized in our hospital, and therefore obtaining these results retrospectively can sometimes be challenging.

# Reviewer report: Reviewer response for version 1

**DOI:** 10.21956/wellcomeopenres.22379.r69858

**Published:** 2024-02-20

**Authors:** Tengku Jamaluddin

**Affiliations:** 1 Department of Medical Microbiology, Faculty of Medicine and Health Sciences, Universiti Putra Malaysia, Serdang, Selangor, Malaysia; 2 Infection Control Unit, Department of Medical Microbiology, Hospital Sultan Abdul Aziz Shah, Universiti Putra Malaysia, Serdang, Selangor, Malaysia

The authors have described the case adequately and provided relevant clinical descriptions, including images depicting the cutaneous involvement of the disease. 

 Discussion pertaining clinical diagnosis explored other possibilities, ie. herpes zoster super-infection etc. Authors aptly justified, and discuss the diagnostic approach of the case. 

 This case will definitely contribute in highlighting awareness on this rare and under-diagnosed disease. 

Are enough details provided of any physical examination and diagnostic tests, treatment given and outcomes?

Yes

Is the case presented with sufficient detail to be useful for other practitioners?

Yes

Is sufficient discussion included of the importance of the findings and their relevance to future understanding of disease processes, diagnosis or treatment?

Yes

Is the background of the case’s history and progression described in sufficient detail?

Yes

Reviewer Expertise:

Infection prevention and control, Antimicrobial resistance, Molecular medical microbiology, One Health, Antimicrobial Stewardship and Diagnostic Stewardship.

I confirm that I have read this submission and believe that I have an appropriate level of expertise to confirm that it is of an acceptable scientific standard.

# Reviewer report: Reviewer response for version 1

**DOI:** 10.21956/wellcomeopenres.22379.r71636

**Published:** 2024-01-23

**Authors:** Yvan Caspar

**Affiliations:** 1 Bacteriology Laboratory, Institute of Biology and Pathology, CHU de Grenoble Alpes, Grenoble, France

This case report presents a rare clinical manifestation of listeriosis in an immunocompromised patient. It is well written and described. Data in low and middle income countries are insufficient which also adds to the significance of this report.

 I only have a few minors queries :

 Introduction section :

 line 3 : replace "Poorly understood" by « insufficiently described » or or state that there are knowledge gaps in low and middle income countries as there is a known burden but which is not well documented.

 Case report section :

-  MALDI-TOF : expand in full letter (except if accepted by the journal guidelines)
-  How long was post-operative antibiotic treatment ?
-  Can you provide more descriptionof the skin lesions ? Were they painful ? pruritic ?
-  a bacterial swab was taken from the pustular rash but the culture is positive from the pus swab.
- I imagine these are the same swabs but may cause confusion for the reader. Was the content of the vesicles clear liquid or pus ?
-  "it is unlikely that any changes to the antibiotic therapy would have altered the outcome in this complex case." I agree with the statement but it may be useful not to presume anything and to delete this part of the sentence as both treatment are effective to treat listeriosis.

Discussion section :

 There is debate about the use of dexamethasone in listeriosis meningitis outcome [1] Was prednisolone stopped during acute infection ? Is there any litterature about the potential effect of prednisolone of the outcome of listeriosis ?

Are enough details provided of any physical examination and diagnostic tests, treatment given and outcomes?

Yes

Is the case presented with sufficient detail to be useful for other practitioners?

Yes

Is sufficient discussion included of the importance of the findings and their relevance to future understanding of disease processes, diagnosis or treatment?

Yes

Is the background of the case’s history and progression described in sufficient detail?

Yes

Reviewer Expertise:

Clinical microbiology, Rapid diagnosis methods, tularemia

I confirm that I have read this submission and believe that I have an appropriate level of expertise to confirm that it is of an acceptable scientific standard.

## References

[1] : Adjunctive dexamethasone treatment in adults with listeria monocytogenes meningitis: a prospective nationwide cohort study. *EClinicalMedicine* .2023;58: 10.1016/j.eclinm.2023.101922 101922 10.1016/j.eclinm.2023.101922 37007737 PMC10050789

# Response

**Published:** 2024-02-29

**Authors:** Terry Evans

**Affiliations:** Nuffield Department of Medicine, University of Oxford, Oxford, England, UK

The authors thank the reviewer for his expert and thoughtful comments that clarify several important aspects of the presentation and treatment of this case.
